# Against the odds: exploring individuals’ pushback mechanisms against commercialized football gambling

**DOI:** 10.3389/fpubh.2024.1325465

**Published:** 2024-04-05

**Authors:** Tunde Adebisi, Ayooluwa Aregbesola, Timilehin Taiwo-Abdul

**Affiliations:** ^1^School of Sport, Faculty of Life and Health Sciences, Ulster University, Belfast, United Kingdom; ^2^Centre for Learning Resources, Timilehin Taiwo-Abdul - Department of Sociology, Landmark University, Omu-Aran, Nigeria

**Keywords:** football gambling, agency, commercialized gambling, gambling marketing, recovery framework

## Abstract

**Introduction:**

The need for money, the pursuit of pleasure, and the liberalized access to gambling have been documented in several pieces of literature as the drivers of gambling. Such drivers are predicated on commercialized gambling, leading to the growth of the gambling industry and constituting a structural influence that normalizes the activity among young people.

**Methods:**

Through a qualitative inquiry, this study investigates the social agentic factors of individuals who are susceptible to gambling. Fifteen non-gamblers were recruited across three commercial cities in Africa, namely Nairobi, Lagos, and Johannesburg. We first established the gambling susceptibility of the participants: their need for money, their passion for football, ownership of a smartphone, access to the internet, and exposure to football gambling marketing.

**Results:**

Consequently, we probed for their agency, which is indicative of why they do not gamble, despite being susceptible to engaging in the activity. Four major agentic factors were identified from the participants; knowledge of the industry’s business model, conserving the integrity of football, identity of self, morality and/or religion model.

**Discussion:**

As such, these factors may be utilized to develop an intervention program for gamblers within the geographical context.

## Introduction

The prevalence of gambling has been predominantly understood from the perspective of the players: Why do people gamble? Some of the few identified drivers include unemployment/underemployment, economic hardship, the pursuit of enjoyment/leisure, peer group influence, etc. (1, 2), all of which excuse the influence of the gambling industry. However, the rapid growth of commercialized gambling could be understood from the stance of the gambling industry: what is driving the expansion of gambling in society? (3). The growing influences in technology, economics, and politics have been identified as contributing to the expansion of the gambling industry (4–8). Drawing on the wide influence of sports content in society, and particularly the popularization and commodification of football (9, 10), the gambling industry is increasingly creating a series of football gambling products that are now being normalized within football (11, 12) and are presented and marketed as an extra activity in the football fandom and a panacea to economic hardship. Similarly, the industry is leveraging several factors to avail their products and services, some of which include indigenous gambling culture, technoliberalism, economic hardship, football fandom, and weak macro-level regulatory regimes, among others (3, 6, 13, 14, 24).

Individuals’ motivation and their ability to gamble involve both their susceptibility and structural influences such as the commercialisation of sports gambling, particularly gambling marketing (15–17), which also involves covert marketing (18, 19), all of which has led to an unprecedented increase in gambling behavior and addictions in recent years (20–24). The gambling industry engages in a series of promotional marketing activities, such as free bets, percentage on deposits, promotional codes, cashback, live cash-outs, football sponsorship, and much more. Such promotions are meant to downplay the downsides or risk perception of gambling, thereby encouraging greater engagement with gambling products and motivating emerging players to continue (25). As such, sports gambling marketing has contributed to the driving factors of sports gambling activities in individuals (21). Such factors have been summarized under three “Ps”: *Poverty*–need for money due to various economic conditions; *Pleasure*–sports fandom, leisure, or pursuit of entertainment; and *Proximity*–liberalized access to gambling products via technology and weak regulations, for instance (3). These represent the structural factors upon which gambling behaviors are predicated.

Social systems, institutions, or activities that wield significant influence and constraint over people’s experiences, including the opportunities that channel individual actions, are regarded as “structures” (26, 27). Structures are neither self-regulating nor autonomous; they are perpetuated and modified by what people do across history (28). Policies further increase the influence of structures, acting as constraints and demanding compliance from individuals within a particular structure. Within the context of the gambling industry, the influence of ‘commercialized gambling’ on gamblers is unprecedented (29–31), and the legitimisation of sports gambling and various liberalized regulations of the same (24, 32) has further contributed to the powerful influence and increasing patronage to the industry. However, as Foucault (33) argues, wherever there is power (influence), there is resistance. Such resistance positions an individual as an actor who reflects rather than responds by reflex to structural influences (27, 34). Individuals with such resistance ability, otherwise known as ‘agency’, can assign meaning to objects, interactions, events, or experiences; define the situation based on those meanings; and then act accordingly. As such, the agency suggests that individuals can oppose structures. For example, it is possible to vote for a preferred candidate during an election despite electioneering or choosing a career/job different from a family business, among others. This ability is hypothesized to be implementable in terms of gambling behavior or otherwise.

## Research objective

Where possible, individual pushbacks against the existing corporate strategies of the gambling industry to market gambling products/services are conjectured to produce one of two things: the first being the ‘coping strategies’–resilience or the ability to negotiate challenges on the part of people who engage in gambling–which sustains their pursuit of new paths to maximize winnings. This usually involves the self-help activities of gamblers that help them minimize or nullify their chances of losing a bet (35). The other is the ‘resistance’ strategy–opposing or building up a defense–that ensures non-participation in gambling activities despite susceptibility to the three ‘Ps’ mentioned above and exposure to the influences of sports gambling marketing. This research examines the latter to understand the human agentive factors, whether convenient or otherwise, that explain the non-gambling behaviors of individuals who are susceptible and predisposed to gamble. This research will first establish participant’s susceptibility to gambling and will consequently probe for the agentive reasons why they do not gamble (or stop gambling), despite experiencing harsh economic crises characterized by poverty or un(der)employment, being a fan of a European football club, and having access to online gambling services via a smartphone and the internet, including several exposures to sports gambling marketing.

### Social agency: resistance to social structure

Social agency refers to the capability of individuals or groups to act purposefully and/or independently to shape their lives as well as the society they live in. Agency is a critical concept in sociology, as it recognizes the importance of individual actions and decisions in shaping social structures and outcomes. The concept of agency is often contrasted with the idea of social structure, which examines the models of relationships, institutions, and norms that influence social life. Anthony Giddens suggests that agency is a necessary condition for social change, as it enables individuals to challenge and transform existing social structures (36). Similarly, Pierre Bourdieu argues that agency is critical for social change, as it allows individuals to contest and transform the dominant symbolic structures and meanings that shape social life (37).

However, the connection between agency and structure is complex and dynamic. While agency can be a form of resistance to social structure, it is also influenced by structure. Social structures may shape individuals’ beliefs, values, and interests, thereby influencing their capacity to act independently. For instance, in Africa, the values and views of religious social structure (particularly Islam and Christianity), which is considered one of the most influential institutions in Africa (38, 39), have identified gambling as an activity related to covetousness, temptation, abuse of resources, and a bad work ethic (40). While the Bible does not explicitly outlaw gambling, the Quran does.

O believers, wine and gambling, idols and divining arrows are an abhorrence, the work of Satan. So keep away from it, that you may prevail. Satan only desires to arouse discord and hatred among you with wine and gambling, and to deter you from the mention of God and from prayer. Will you desist? (Qur'an 5: 90–91)

Similarly, the Christian faith, particularly the Conservative Protestants, argues against many gambling types based on scriptural passages and social facts. Gambling is considered idolatrous and contradictory to the omniscience of God. Devoting oneself to luck or chance is like worshipping heathen gods. It is also argued that gambling contributes to the primacy of material gains in relationships. Similarly, the work ethic that runs through the Bible is broken by gambling. A biblical working ethic opposes quick-wealth schemes that are part of the charm of gambling. Similarly, gambling is considered addictive, encouraging Christians to always be cautious and self-regulated (41).

Although gambling is largely condemned by these monotheistic religions, ‘Folk’ or traditional faiths, such as indigenous religions, often have a positive view of gambling precisely because of the elements they have in common, including representations of gambling in their rituals, magic, and myths, and the spiritual dimension of gambling (42–44). Hence, devotees to these religions could be influenced by adopting such religious beliefs as theirs and shaping their values and interests (or otherwise) as it relates to gambling.

The concept of human agency is essential for grasping the role of individuals and groups in shaping their social life. While social structures can constrain individual agency, agency can also be a form of resistance to social structure, creating new possibilities for social change. By recognizing the importance of agency, we can better understand the dynamic interplay between individuals and society and the potential for social transformation.

### Method

In the light of the focus of this study on the agency factors of non-gambling, a qualitative research design is appropriate. Qualitative research enables a deep understanding of individuals’ subjective experiences in the social worlds they inhabit and allows the researcher to interpret the meanings they attach to particular social practices (45). Qualitative research methods are wide-ranging, but this study adopts a phenomenological research design to collect and analyze the unique narratives of participants.

### Research sample

Participants in this study are divided into two categories: those who do not gamble and those who stopped gambling, despite being predisposed and influenced to gamble. The study employs a three-stage sampling technique. In the first stage, South Africa, Nigeria, and Kenya are purposefully selected as the locales for this study due to their high and increasing population of gamblers, expenditure on gambling, and football fandom. These countries are considered the three biggest gambling markets in Africa (3). In the second stage, each country’s commercial capital, Johannesburg, Lagos, and Nairobi, is selected. Moreover, each city is cosmopolitan, attracting a diverse population in terms of ethnicity, religion, and culture. In the third stage, 15 participants, 5 from each city, were selected at football viewing centers during live European football matches.

### Participant recruitment

Participants were recruited from various football viewing centers, where individuals gathered in various social arrangements to watch live European football matches. The researchers approached potential participants following an initial observation and informal conversation during such live matches. The snowballing technique was used subsequently to recruit other participants. The key recruitment criteria for participants in this category are persons of 18 years or older who watch and support a team in any of the ‘top’ European leagues, have a smartphone and stable access to the internet, as well as basic knowledge of sports gambling, and are unemployed/underemployed. Basic knowledge of sports gambling is as crucial as every other inclusion criterion, and this is measured via participants’ exposure to sports gambling marketing, which is expected to aid their understanding of football gambling products, markets, and brands.

### Data collection

Data were collected via observations, informal conversations, and semi-structured interviews that probed for agentive factors among non-gamblers in the selected cities. Observations and informal conversations aided the third stage of the sampling procedure to recruit the appropriate participants for the study. Semi-structured interviews were consequently conducted, which were recorded with the aid of a voice recording app and later transcribed for analysis.

### Data analysis

The data were analysed using phenomenological analysis, which involves exploring and describing the essence of a phenomenon as it is experienced by individuals. It typically involves a detailed examination of individual experiences and perceptions and the identification of common themes and patterns across such experiences (46, 47). The analysis followed the following steps:

1. Familiarization with the data: we became familiar with the data by reading and re-reading transcripts from the interviews.
2. Identification of significant statements: we identified significant statements or phrases that capture the essence of the phenomenon being studied.
3. Grouping of significant statements: we then began to group the significant statements into themes that reflect the essence of the phenomenon.
4. Development of a description: we developed a description of the phenomenon based on the themes identified in the previous step.
5. Validation of the description: it involves the validation of the description by checking it against the data and ensuring that it accurately reflects the experiences of the participants.

### Summary of methodology

**Table:** 

City/country	Johannesburg, South Africa	Lagos, Nigeria	Nairobi, Kenya
Sampling	Three-stage sampling: purposive, stratified, and snowball		
Sample size	15; 5/city		
Research location	Football viewing centers		
Data collection	Participant observation, informal conversation, and semi-structured interviews		
Data analysis	Phenomenological analysis		

### Ethical considerations

All participants received an information sheet before the interview and had the opportunity to discuss the nature of the project and any concerns that they may have. Every participant who agreed to proceed with the interview was required to verbally consent to the interview and sign a consent form. All interviews were recorded, and the interviewees were allowed to review the interview transcript to ensure accurate representation. The recordings were later deleted after the transcripts had been vetted by the participants. Consequently, the identities of the subjects were protected. Ethical clearance was obtained from the Landmark University Center for Research, Innovation, and Development (LUCRID).

### Results

The focus of this study enables us to identify 15 individuals within ‘legal age’, across three cities in Africa, who are influenced and/or predisposed to gamble but do not gamble or have stopped gambling.

### Sociodemographic factors

Five key demographics, such as sex, age grade, religiosity, level of education, and employment status, were considered important to put individual narratives in context. This is presented in Table 1.

**Table 1:** Sociodemographic factors of participants.

No.	City	Sex	Age range	Religion	Education	Employment
1	Nairobi	Male	Late 20s	Christianity	Bachelors	Underemployed
2	Nairobi	Female	Late 20s	Christianity	Bachelors	Underemployed
3	Nairobi	Male	Late 20s	Christianity	Bachelors	Underemployed
4	Nairobi	Male	Early 30s	Islam	Bachelors	Unemployed
5	Nairobi	Male	Early 30s	Christianity	Masters	Underemployed
6	Lagos	Male	Late 30s	Islam	PhD	Underemployed
7	Lagos	Male	Early 30s	Christianity	Masters	Underemployed
8	Lagos	Male	Early 20s	Christianity	Bachelors	Unemployed
9	Lagos	Male	Early 30s	Christianity	Masters	Unemployed
10	Lagos	Female	Early 20s	Christianity	Bachelors	Underemployed
11	Johannesburg	Male	Early 30s	Christianity	Bachelors	Underemployed
12	Johannesburg	Male	Almost 20	Islam	High education	Unemployed
13	Johannesburg	Male	Late 20s	Christianity	Basic education	Unemployed
14	Johannesburg	Male	Late 20s	Christianity	Bachelors	Unemployed
15	Johannesburg	Female	Early 30s	Christianity	Masters	Unemployed

#### Sex

Using the multi-stage sampling technique described above, we wanted to interview both male and female individuals. However, the latter was difficult to recruit and made up only 20% (3/15) of the sample, which reflects the position of several previous studies in Africa, placing the male folks at the core of the gambling scene (1, 35, 48).

#### Age (range)

The interconnectedness between age and agency has been established and discussed in several pieces of literature (49–51), which refers to how individuals’ abilities to act and make choices are influenced by their age. To avoid age-cheating, a concept that explains the (actionable) deliberate attempt of Africans to conceal their real age (52, 53), we adopted a decolonising research orientation where participants were asked to provide an age range instead; ‘Late 20s’ and ‘Early 30s’ have an equal share of 66.7% (10/15).

#### Religiosity

In Africa, religion plays important roles, both as a structure and as one that contributes to human agency (38, 39, 54). The religious affiliation of the participants is skewed toward (Pentecostal) Christianity. However, using Rule’s (54) model, the frequency of attendance at religious services or meetings, the orthodoxy of beliefs concerning Biblical teachings, and religious denominations, we discovered low religiosity among the research population.

#### Level of education

The interconnection between the level of education and agency refers to how individuals’ educational attainment, or their level of formal education, can influence their capacity to act and make choices in various domains of life (55). Although snowballing was employed in the third stage of the sampling technique, we sought to recruit individuals with basic or no education when we had enough participants with tertiary or high school education. However, the former group was difficult to recruit. Most participants expressed no knowledge of or affiliation with persons with such a low level of education and did not gamble or had stopped gambling.

#### Employment status

The economic status of individuals is one of the inclusion criteria for this study. Probing individuals who are unemployed or underemployed fulfills the criteria of who may be experiencing economic hardship and, thus, are susceptible to gambling. These particular data reveal 53.3% (8/15) of underemployed individuals and 46.7% (7/15) of unemployed individuals.

### The structural factors that predicate gambling behaviors

We probed for the structural factors upon which gambling behaviors are predicated, using Bitanihirwe et al.’s (3) summary of the drivers of gambling: *Poverty*–need for money due to various economic conditions such as unemployment and underemployment; *Pleasure*–football fandom; and *Proximity*–liberalized access to online gambling products via the use of a smartphone and internet connection. Additionally, we explored participants’ exposure to sports gambling marketing, which aids their knowledge of football gambling products, markets, and brands. These were done to establish susceptibility to football gambling among the participants.

### Poverty–economic crisis characterized by unemployment and underemployment

Economic hardship is a major structural influence upon which gambling behavior is predicated among most gamblers in Africa (1, 56) and elsewhere (57, 58). In the context of this study, poverty, as a structural influence, is operationalized in terms of unemployment and underemployment. We, therefore, probed for participants’ current economic status and what they would consider ‘a dream job’ and ‘a fair pay’, which will not only cushion economic hardship but also avail them of a ‘comfortable’ life. Table 2 presents participants’ ideal employment and monthly earnings that will, at least, suffice to meet everyday needs. This is done to establish that participants are currently low-income earners and, hence, susceptible to gambling.

**Table 2:** Participants’ current status and proximity to expected economic status.

No.	Current status	Dream job	Fair pay (£)
1	Underemployed	Financial officer	Money is never enough
2	Underemployed	Statistician	357
3	Underemployed	Digital marketing	594–713
4	Unemployed	Naval officer	Whatever they earn
5	Underemployed	Nursing	1,189
6	Underemployed	Director in my field of discipline	Money enough to meet both immediate and future needs
7	Underemployed	Academia	348 upward
8	Unemployed	Remote jobs	2,610–3,480
9	Unemployed	Lecturing	261, 296
10	Underemployed	UX designer	896 at least
11	Underemployed	Civil engineer	As much as I can earn on the job
12	Unemployed	Football player	200,000
13	Unemployed	Music artist	So much money
14	Unemployed	Software engineer	Plenty of money
15	Unemployed	Pilot	Enough to set me up well

The result shows that both divisions of ‘poverty’ are virtually the same: underemployed (53%), and unemployed (47%), as shown in Tables 1, 2. While participants reported a variety of dream jobs, fair pay may be categorized under two themes: a definite amount and non-finite.

### Pleasure–(club) football fandom

In the context of this study, fandom in football is operationalized in terms of the meaning attached to football, favorite European football club, year(s) of support, reason(s) for support, and expectation(s) of such club. The response to ‘favorite European football club’ and ‘years of support’ is definite, while responses to other indicators are categorized into themes. Most people support Chelsea Football Club (8/15), Arsenal Football Club (3/15), Manchester United Football Club (3/15), and Liverpool (1/15). The least and most years of support for such clubs are 7 and 25 years, respectively.

The theme emerging from participants’ narratives about their (other indicators of) football fandom is presented in Table 2.

All participants confirmed their affiliation with football, particularly with European club football. The meanings attached to football are captured under three themes: football as a culture, as entertainment, and as a commodity. Similarly, the reasons for supporting a particular football club include the style of play adopted by a European football club or current coach and the set of values propagated by the club. Third, participants’ expectations of the club, particularly at the start of a new season/competition, were captured as ‘winning trophies’, and ‘good football’. Some of the narratives are presented in Tables 3, 4.

**Table 3:** Themes and coded descriptions of three indicators of football fandom.

Indicator	Theme	Code description	Cases
Meaning attached to football	Culture	An entity that bounds people together irrespective of inherent social belonging	6/15
	Entertainment	A leisure activity for past-time and relaxation	7/15
	Commodity	A means to sell many other products/services	2/15
Reason for support	Style of play	Comprises of formation, and the pattern of play adopted by a club or a coach	3/15
	Pan-Africanism	Support for African players playing in European football clubs	10/15
	Philosophy of the club	A set of values promoted by a club, particularly the academics and the grooming of young players	2/15
Expectations on such club	Winning trophies	Winning mentality is an important factor; plenty of goals, a winning streak, and silverware	11/15
	Good football	Entertainment is an important factor; the style of play, the caliber of players, and the extra-football activities within the club	4/15

**Table 4:** Some narratives on three indicators of football fandom.

Indicators	Themes	Narratives
Meaning attached to football	Culture	So, it’s a culture. When it comes to football, you know, particularly when you see someone who belongs to the same club that you support, you can hardly see or perceive things that make us different; race, culture, religion, or status. It kind of erases all these differences, you know. It’s one of those few things that bring people together regardless of their differences. (Lagos/Male/Early 30s/Masters/Unemployed)
	Entertainment	I see it as entertainment and something you do for leisure. (Nairobi/Male/Late 20s/Bachelors/Underemployed)
	Commodity	It’s a source of income. What I really meant as a source of income … you know we sell merchandize like jerseys and we do football news which still generates money and we engage in (like) some small tournaments in which we get sponsorship to get some cash. (Johannesburg/Male/Early 30s/Bachelors/Underemployed)
Reason for support	Style of play	I think, because of their style of play. Okay. The style of play is, you know, from Pep Guardiola’s Barcelona … the tiki-taka style of play is what I really like. I like, you know, possessive, the possessive football kind of thing. That’s why, even now, I see myself still leaning toward Manchester City, in the Premier League because of Guardiola … but still, Barcelona is still my main club … (and) it should be close to a decade now. (Lagos/Male/Early 20s/Bachelors/Unemployed)
	Pan-Africanism	For Chelsea basically, the way they support African players. Talk of John Obi-Mikel, you can talk of Drogba, you can talk of Essien. It’s the way they support African players. (Nairobi/Male/Late 20s/Bachelors/Underemployed)
	Philosophy of the club	Majorly because of their philosophy of play under Arsene Wenger. He had a system, for instance, the back four were to defend as a unit. However, he allows individual players to use their prerogative in spite of his system. (Lagos/Male/Late 30s/PhD/Underemployed)
Expectation on such club	Winning trophies	To banter with friends and the hope to do better like winning the league after a long time. (Lagos/Male/Late 30s/PhD/Underemployed)
	Good football	You see, Apologies to Chelsea fans, one of the reasons why I do not like that team, because, you know, to me, football is more than just to score; 5–0, 6–0 … it does not really … I want to see you play a beautiful game … the rhythm, the flow, the passes, the dribbles are the things that make a football, not just shooting the ball from the post, you know, long shots here and there. Arsenal plays good football. (Lagos/Male/Early 30s/Masters/Underemployed)

### Proximity–liberalized access to online gambling products via the use of a smartphone and internet connection

As a structural influence, proximity, in the context of this study, refers to easy access to online gambling *via* the use of a smartphone and internet connection. Table 5 presents participants’ responses to their access to smartphones and the internet.

**Table 5:** Smartphone and internet access.

Smartphone and internet access	Smartphone brand	Level of use	
		Smartphone usage experience (years)	Frequency of internet use on smartphones
Nairobi	Nokia	8	20 h/day
	Techno	10	6 h/day
	Samsung	9	24 h
	Techno	9	12 h/day
	Samsung	8	24 h
		
Lagos	Samsung	12	24 h
	Samsung	5	6–8 h/day
	Apple	10	24 h
	Techno	12	10 h/day
	Techno	1½	24 h
		
Johannesburg	Samsung S21 plus	16	24 h
	Huawei	2	24 h
	Samsung	10	16 h/day
	Apple	5	24 h
	Samsung	10	8–10 h/day

Access to smartphone and Internet services was examined in terms of smartphone brand, smartphone usage experience, and frequency of Internet use on participants’ smartphones. Inquiries about access to smartphones and the Internet were guided by the following questions: “What brand of smartphone do you own?” How long have you had a smartphone? and “How often do you have internet service on your smartphone?” More participants (7 each; 46.7%) reported that they own Samsung phones than those who reported possessing Techno 4 (26.7%), Apple 2 (13.3%), Huawei 1 (6.6%), and Nokia 1 (6.6%).

Experience of usage was measured by asking the participants how long they had been using smartphones. Table 5 reveals the experience participants have with using smartphones. The results indicate that most of the participants (11 each; 73.3%), began using smartphones on or before 2015 (8 years and older). However, fewer participants (2 each) had a lesser experience of 5 years and 1–2 years of smartphone usage, respectively.

The frequency of Internet use on smartphones was examined by asking the participants the following: “How often do you have Internet service on your smartphone?” The responses from the participants were categorized as follows: always (16–24 h per day), often (10–15 h per day), and sometimes (6–9 h per day). As shown in Table 5, more than half of the participants (10 each; 66.7%) always use the Internet on their smartphones. Three participants (20%) reported using the Internet on their smartphones often per day. Fewer participants (2 each; 13.3%) reported that they sometimes have Internet service on their smartphones.

### Knowledge of football gambling via sports gambling marketing

We explored participants’ exposure to sports gambling marketing, which aids their knowledge of football gambling products, markets, and brands. Participants were asked the following questions: “What do you know about football gambling?”–This is an inquiry to probe for individuals’ basic or otherwise knowledge about football gambling. “How did you learn about football gambling?”–This is an inquiry to probe of participant’s source(s) of knowledge about football gambling, and “How often do you see gambling-related ads in a day?”–This is to probe for participant’s level of exposure to football gambling.

The theme emerging from participants’ narratives about what they know about football gambling is presented in Table 6, while their sources of knowledge, as well as their level of exposure to football gambling, are presented in Table 7.

**Table 6:** Participants’ knowledge of football gambling.

Indicator	Theme	Code description	Cases
Knowledge of football gambling	Gambling markets	Participants’ description of the activity via gambling markets such as over 2.5, goal–goal, first team to score, etc.	5/15
	Gambling brands	Participants’ description of the activity via gambling brands such as Sportpesa, Betway, Bet9ja, etc.	6/15
	Game of scam	Participants’ description of the activity as a game that promises one thing but delivers another.	2/15
	Game of hope	Participants describe the activity as a game that offers people the hope to win big, depending on their knowledge of football.	2/15

**Table 7:** Participants’ sources of knowledge about football gambling and level of exposure.

No.	Sources of knowledge	Level of exposure
1	Advertisement, and you know we have friends who gamble and we meet, and maybe you are watching football with somebody who is gambling, so you get to know about it	More than 5 times in an hour
2	Through TV adverts	At least once a day
3	My first contact with gambling ads is in the Newspaper, and that was when SportPesa came to Kenya. Today, the ads are all over the place; in the streets and of course online.	All the time
4	The avert is everywhere. And you know, we meet people who play (gambles) every day	Everyday
5	I get to see these adverts when I surf the web on my phone.	24/7
6	Through informal conversations with colleagues at work and neighbors at home. Also, as I surf the internet, during live matches, and on some merchandize	Very frequently
7	The way football gambling is spreading so fast, it’s like wildfire. You hardly see someone around you that is not into it. So while they are talking about it, I’m learning new things about gambling. Asides from hearing from people’s personal experiences, gambling, generally, when you watch football matches, particularly on Super Sports, you know, the advert is right there, right in front of you. Also, when you want to check something online, even when what you want to check is not about gambling, it (football gambling ads) just pops up in your face. On social media, it’s everywhere as well.	I can see it every time, every day that I want to watch a football match. It’s always there
8	It’s basically my friends. I’ve seen the platform occasionally but I’ve not sat down to say… okay I want to (bet). I have some of them come to me because I do not bet. So my insights, to them, will be from a neutral ground… since my own money is not involved, They feel I’m not pressured to overthink it. So they’ll just come, ‘Okay, (name mentioned), what did you think of this match? I see adverts about their (the betting brands) sites as well’.	While surfing the internet, and when watching a Live game.
9	I have friends who are still actively involved, even though I’m trying to put them out. (Also) when I’m browsing, ads pop up, especially Bet9ja ads. It used to be Nairabet ads. I have never responded to the ads, because I did not understand gambling until someone showed me and put me through how to bet	Some minutes ago I got a pop-up ad from Bet9ja. I see that every day
10	Since I was in secondary school, I think my first conscious knowledge of it would be around SS1 (First class at the Senior High School), but I do not know. But I have classmates who used to stake every weekend and then they would come to class on Monday and tell us, okay, this is how much I (won) … I know someone who did that and paid his fees. So it’s not like I’m knocking it like it’s too evil or something. But basically, that’s the knowledge. That’s how the knowledge started. And then along the way you get to make friends who would post stuff on social media. You also run into it. There are Twitter accounts that are dedicated to posting ‘sure odds’, you know, stuff like that. And then also influencers that always put it in your face.	I do not encounter it often when browsing the internet because that’s not anything I would search for. But when watching skits, I do that a lot to unwind. It always comes up
11	As long as you own a smartphone, you get to learn about these things through pop-ups, advertisements, celebrity endorsements, influencers, friends, and everything. But for me in particular, and where I can relate, and (what) draws my attention the most are through my friends	Almost all the time
12	As a football player, I used to hear team players and opponents alike talk about sports betting, Then, you hear them say, straight win, over 2.5 goals … ‘What is this all about’?	I see it all the time, you cannot escape it
13	My friends told me about it, you know	Every time bro
14	I see the online adverts, and even during live games	Every single day
15	Someone gave me one of their T-shirts	Very common

More participants (6 each; 40%) described their knowledge of football gambling in the light of sports gambling brands. It appears that the saturating amount of sports gambling brands in African markets makes football betting readily recognizable. A participant narrates:

I know there are lots of betting brands everywhere. There are much more than we used to have a decade ago. The likes of 1XBet, BetKing, and Sporty Bet are somewhat new. We used to have the likes of NairaBet, 1960Bet, Winners Golden Bet, and Bet9ja dominating the football gambling scene in Nigeria. (Lagos/Male/Late 30s/PhD/Underemployed)

Another participant who once gambled gives a similar perspective:

I started first with Nairabet, then Bet9ja came, then bet with Bet9ja for a couple of times. And then BetKing came. Today we have a lot of them; the likes of SportyBet, MSport, 22Bet, 1XBet, BetWG, yeah, those are just the ones I can actually recall for now … and MerryBet. (Lagos/Male/Late 20s/MSc/Unemployed)

Quite similarly, five participants (33%) reported their knowledge of football gambling by describing various gambling markets available for players. Interestingly, the source of this knowledge about gambling comes from peers whom they have heard mention or describe some of these gambling markets. For instance, a participant narrated as follows:

About gambling, I know some gambling fans. I have an idea of how somebody can gamble … yes yes I know they talk about, maybe over 2.5, under 2.5, goal-goal, the first team to score, which player will score. (Nairobi/Male/Late 20s/BSc/Underemployed)

Understanding football gambling as a game of scam and a game of chance contributes equally to 27%. The narratives are presented, respectively.

I know it is now a thing. It is very popular with everyone because they (the betting companies) always tell you that you can win. However, I haven’t seen many people who have truly won. I think they (the betting companies) rely on so many losses to fund a single win. (Johannesburg/Male/Early 20s/High Education/Unemployed)

Okay, for me, football gambling is a platform where, you know, persons who are passionate about football can be rewarded for their knowledge and be confident about their knowledge. (Lagos/Male/Early 30s/MSc/Underemployed)

Participants reported their knowledge sources for football gambling. Different forms of advertisement were prevalent; they included both online and print adverts in the form of pop-ups on their smartphones, printing on newspapers, and merchandize. Live TV/match adverts were also reported. Social media handles (particularly Twitter) and posts also serve as sources of information for individuals. Some Internet influencers and celebrities alike use their social media platforms to promote football gambling brands to their audience. While some participants narrated how they had been introduced to football gambling by their peers, a few who had once gambled (but have now quit) were introduced to the activity by their friends.

### The agentic factors that discourage gambling behaviors

Despite these levels of exposure to football gambling marketing and susceptibility to football gambling, characterized by ‘poverty, pleasure, and proximity’, the agentic factors of participants were probed. Participants in this study are broadly categorized into two groups: those who do not gamble, and those who have stopped gambling. More participants (12 each; 80%) reported that they had never gambled than three participants (20%) who reported that they had stopped gambling. This result, particularly the low population of recruited participants who have stopped gambling, is indicative of the complex process of recovery from gambling addiction and that such addictive activity is better not plunged into.

The theme emerging from participants’ narratives about agency against football gambling is presented in Table 8.

**Table 8:** Themes of participant’s agency against football gambling.

Indicator	Theme	Cases
Never gambled	Knowledge of the industry’s business model	3/15
	Conserving the integrity of football	2/15
	Identity of self	6/15
	Morality and religion	1/15
Stopped gambling	Morality and religion	2/15
	Identity of self	1/15

Four major agentic factors were identified from participants’ narratives of why they do gamble, as it were: (1) knowledge of the industry’s business model; (2) conserving the integrity of football; (3) identity of self; and (4) morality and/or religion.

### Knowledge of the industry’s business model

Subjective accounts that project football gambling as a negative activity probe the industry business model. Such interests serve as a basis to rationalize individuals’ involvement with football gambling. Moreover, it similarly gives them an idea of what kind of person they will become if they patronize such an industry. A participant questioned the financial commitments of the gambling establishment, and how they make maximum profits:

I think about how economical are they, (in terms of) how they can improve one’s financial status, and from that point I cannot gamble because gambling firm has employees. So, how are they paying the employees? Somebody has to bet or do the gambling for them to give the money and pay their employees. I've seen them saying ‘I've made huge profits’, like some like a million dollars. That's a lot of money. And in my mind, when I gamble, I'm giving somebody money that they haven't worked for hoping to get more money. it’s wasting your resources which can be channeled into another thing that can make more economic sense and make more money, or even if you're making less money, it’s something that is viable, it’s not like luck. You can explain, you can literally explain it. (Nairobi/Male/Late 20s/BSc/Underemployed)

Similarly, a participant, who has recovered from gambling probed the morality of the industry’s business model. He asserts that football fans are naturally inclined to predict the outcome of football games and that betting companies are only taking advantage of fans’ intrinsic activities when they enjoy their favorite sports:

Naturally, as a football fan, we’re gamblers. Before you place your money to bet, we gamble, and we do this through predictions. The moment we predict the outcome of an event, and in this case football, we are already, you know, diving into gambling. I stopped because of what I now know about the industry. It’s enough to say that they target vulnerable people. I have to constantly remind myself of what I know about the game, whenever I feel the need to gamble, then and there I get turned off by it. (Nairobi/Male/Late 20s/BSc/Underemployed)

While some expressed clarity as to how the industry works, others speculated. However, such speculations are enough to form an agency against the activity.

Gambling negates everything I believe in. They tell you to try again and again when you don’t win. I think I can say it’s from a place of contentment. Yeah. And then not really being greedy in a way. And then I'll say I feel gambling is not sustainable. Because, I always think, of course, I see people win a lot of money online, and offline. So when that happens, their orientation is that okay, once they’ve won this amount of money, if anything happens, maybe they exhaust it (the winnings), they’ll come back again. And it doesn’t work that way, because, you can win millions today and then not win for the next two years. I think the industry has something in place to make sure of that. So it is not really sustainable to me. It’s not like I've not thought about it. Every time the thought comes to my mind, I feel I have already lost the money. So, it doesn't just happen. (Lagos/Male/Early 20s/BSc/Unemployed)

### Identity of self

In other submissions, participants expressed self-consciousness and the willingness to maintain their self-image. Moreover, such participants believe that those who gamble are susceptible to other social vices.

I think gambling is predicated on luck. I believe it is not a wise way to income generation. I cannot waste my limited resources on such a thing. Most importantly, particularly before the prevalence of online betting, when people need to place their bet in a betting shop, I never fancied the idea of going into such venues, because of my self-image and the kind of persons who patronize gambling; such persons are suspectable to criminality and I don’t want to be associated with such. I said that because, you know, vices breed vices. When you’re in the midst of those who gamble, you can be easily introduced to similar activities; like, internet scams, theft, forgery, and all sorts of money-related vices. (Lagos/Male/Late 30s/PhD/Underemployed)

A perspective from a female participant is presented below:

First of all, I’ll say upbringing. It's not something I ever saw my parents do. Okay. My brothers, no matter how much they love it (football), it's not something they would do. So I mean, I would go to school and see people doing that, but like home is like the first point of contact. So it's just not something I saw them doing. And even the people I call role models in my life. Like I just didn't grow up seeing them do that. And then as you know, I began to get my own agency and form my own opinions about the world. I just saw it as, I mean a waste of time, first of all, I think it can give you high blood pressure (laughs). Actually, I see it as being irresponsible … that you stake money on something so unpredictable. You're spending your time looking for odds, joining groups, and trying to know how to predict. You know? I will feel so stupid, and I feel those who have such a routine should reconsider. I think they're just more stable, sure, honest ways to make money. You know, putting a lot on the outcome of something that you cannot predict. I'm generally a risk-averse person though … I've also never done any of these Ponzi schemes. It makes anyone look so stupid. (Lagos/Female/Early 20s/BSc/Underemployed)

### Morality and/or religion

Part of the self-image narrative is the place of religious morality in building up agency against football gambling. As narrated by a former gambler, “Christian conscience” set up a path of recovery from a football gambling addiction:

When I was on my I.T. in Lagos, I spent lots, a whole lot of money on dog races, different things, virtual, real-time football, and all of these things. And I (started) listening to some messages, some Christian messages, and like okay, ‘gambling is not good’. And the Pastor I follow very well, preached against betting. And that was when my Christian conscience or let me say my religious belief was now staring me in my face. I'm looking forward to my PhD and trying to just do things and impact my environment. I get busy with academics, my church, also my family actually. I just bring those things in between, if those things (the urge to gamble) want to come. I just put my family, my church, and probably the work I have to do; I just put them in between to create that boundary between me and betting. (Lagos/Male/Early 30s/MSc/Unemployed)

Similar to the previous submission, another participant alluded to religiosity as an agency that contributes to his non-gambling behavior.

I don’t really believe in gambling, and I don’t want to limit it to a spiritual aspect, you know. As a Christian, you’ll be told, don’t gamble. I just believe that one of the reasons why I don’t really see gambling to be too productive is that it can be very addictive. And most things in life that are very addictive always have a downside. (Johannesburg/Male/Early 30s/BSc/Unemployed)

### Conserving the integrity of football

In other submissions, participants’ agency against football gambling was formed through their passion and quest to protect the integrity of the sport. One participant described match-fixing as a consequence of football gambling:

I don't gamble because gambling causes match-fixing. It’s the main thing that causes match-fixing in the football world … so I don't gamble because if I gamble I'm promoting that. (Johannesburg/Male/Early 20s/High Education/Unemployed)

Similarly, another participant submitted:

Football is called the beautiful game. To be honest, for the sport to remain beautiful, it should be void of things like gambling. I am one of those people who believe that we don’t have to bet on a football game before it becomes enjoyable. (Nairobi/Female/Late 20s/BSc/Underemployed)

## Discussion of findings

Five key demographics–sex, age range, religiosity, level of education, and employment status–including the cities–were used to contextualize the narratives of the participants for this study. Female folks are only 20% of the sampled population, which is reflective of the position of several previous studies in Africa, placing the male folks at the core of the gambling scene (1, 35, 48). The interconnectedness between age and agency has been established and discussed in several pieces of literature (49–51), which emphasizes the ability of individuals to act and make choices with respect to how advanced they are in their chronological age. In this study, more participants (66.7%) are between their late 20s and early 30s. The religious affiliation of the participants is skewed toward (Pentecostal) Christianity. However, adopting Rule’s (54) model of measuring religiosity, particularly in African society, religiosity was found to be low among the sample population. Although snowballing was employed at the third stage of the sampling technique, we sought to recruit individuals with basic or no education when we had a saturation of participants with tertiary or high school education (86.6%). The former group was difficult to recruit. Most participants expressed no knowledge or affiliation with persons with such a low level of education and did not gamble, as it were. This is indicative of the significance of education in agency (55). Finally, the study purposively sorts for unemployed and underemployed individuals to fulfill the criteria of those who may be experiencing economic hardship, thus making them susceptible to gambling.

In this study, susceptibility to participating in football gambling is the prerequisite in probing for individuals’ agency against football gambling. Through participant observation and informal conversations, we were able to deduce susceptibility; however, an in-depth interview was adopted to establish susceptibility. The identified structural factors that make people predisposed or susceptible to engaging in football gambling have been captured under Bitanihirwe et al.’s (3) three ‘Ps’: Poverty, Pleasure, and Proximity. Essentially, because our sample populations are non-gamblers, their knowledge of football gambling was also probed in order to establish their level of exposure to sports gambling marketing.

*Poverty* is operationalized as an economic crisis characterized by unemployment (47%) and underemployment (53%). Low-income earners (underemployed) like those in this study have been reported to be the most prone to gambling (57–59). Similarly, studies have shown that poverty, characterized by unemployment, increases the chances of problems and at-risk gambling (60, 61). Hence, participants in this study are not only susceptible to gambling but also to problem gambling.

*Pleasure* is operationalized as club football fandom. Several indicators were used to establish individuals’ fandom for football: the meaning they attached to the sport, their favorite European football club, years of support, reasons for support, and expectations from the club. The result from this section suggests a vibrant European (particularly English) football fandom among the participants. This is consistent with several studies in the continent that reveal the affiliation of African football fans to European football clubs at the expense of their country’s domestic leagues (4).

*Proximity* refers to the liberalized access to online football gambling *via* the ownership of a smartphone and Internet service. The impact of *proximity* is unprecedented, as reported in several studies (3, 30, 62). A majority (60%) of the participants in this study are owners of two of the most-ranked smartphones in the world: Samsung and the iPhone. According to a market survey, Internet surfing ranks highest in the frequency of using smartphone functions, and the Samsung brand was the top-ranking performer, with Apple being the second, in customer satisfaction (63). This indicates that, although all the participants own smartphones and have constant internet service on such phones, more of the surveyed population experiences better efficiency in terms of internet surfing. Easy access to gambling *via* the Internet has been reported to contribute significantly to the prevalence of football gambling in Africa (64).

Since the study populations are non-gamblers, it became necessary to explore and establish their knowledge of football gambling, despite their susceptibility to the activity. This is done by probing for participants’ exposure to sports gambling marketing, which aids their knowledge of football gambling products, markets, and brands. There are several avenues of exposure to football gambling, including social interactions and various forms of advertising, which have been highlighted in various studies (12, 65–67). Overall, the level of exposure to the different sources of football gambling marketing is reported to be high. It has become part of participants’ daily experiences, especially when they are enjoying their pastime activities, such as watching a live football match, surfing the web, or watching internet skits.

In this research, four reasons why people do not gamble, as it were, have been identified. These reasons inform the processes of their behavior, perception, and identity of self (68), which form their agency against football gambling. For example, knowledge of the gambling industry’s business model serves as a basis to excuse some from the activity. Similarly, the identity of self encourages individuals to preserve their self-image. Participants expressed self-consciousness and a willingness to maintain their self-image. They believe that those who gamble are susceptible to other social vices, such as internet scams, theft, forgery, and all sorts of money-related vices. The impact of religiosity is minimal in everyday cosmopolitanism (69); however, for a relatively few participants, religiosity acted as a threshold for forming an agency against football gambling. Participants in the study also expressed their passion and quest to protect the integrity of football. Match-fixing has been described as a consequence of football gambling; as such, the beautiful game should be void of things like gambling.

## Conclusion

Knowledge of the industry’s business model, conserving the integrity of football, the identity of self, and morality and religion are the identified reasons why people do not gamble or stopped gambling. As such, these factors can be utilized to develop an intervention program or recovery framework for gamblers who desire to quit the activity. Participants who had a clear understanding of the industry’s business model viewed gambling as a negative activity that targets vulnerable individuals. In this context, the vulnerable are considered to be the less informed–it appears that formal education plays a role–and every other factor that makes one susceptible to gambling. Findings from scholarly articles about the structure of the gambling industry, their corporate strategies, and business models should be transcribed into lay understanding and disseminated on platforms that are assessable and resonate with the public.

This study also reveals that those who were concerned with their self-image did not gamble to avoid association with those who engage in criminal activities. Understanding these factors can help develop interventions to reduce the prevalence of football gambling in society. Interventions can be designed to provide ingenious grassroots gambling education to individuals with a clear understanding of the industry’s business model and the risks associated with gambling; such education programs can highlight the negative impact of gambling on individuals’ self-image and religious and moral beliefs, which can be used to discourage the activity. Similarly, addressing gambling at football events can discourage match-fixing. Reducing problem gambling can promote healthy social values and improve the overall wellbeing of individuals and society.

## Data availability statement

The raw data supporting the conclusions of this article will be made available by the authors, without undue reservation.

## Ethics statement

The studies involving humans were approved by the Landmark University Center for Research, Innovation, and Development (LUCRID). The studies were conducted in accordance with the local legislation and institutional requirements. The participants provided their written informed consent to participate in this study. Written informed consent was obtained from the individual(s) for the publication of any potentially identifiable images or data included in this article.

## Author contributions

TA: Conceptualization, Data curation, Formal analysis, Investigation, Methodology, Project administration, Resources, Supervision, Validation, Visualization, Writing – original draft, Writing – review & editing. AA: Data curation, Formal analysis, Investigation, Supervision, Writing – review & editing. TT-A: Investigation, Project administration, Writing – review & editing.
